# Deciphering Microscopic Agglutination Test (MAT) Serogroup Cross-Reactivity in Leptospirosis: The Influence of Age and Antibody Titers

**DOI:** 10.3390/tropicalmed10100275

**Published:** 2025-09-24

**Authors:** Eric J. Nilles, Cecilia Then Paulino, William Duke, Ronald Skewes-Ramm, Adam Kucharski, Colleen L. Lau

**Affiliations:** 1 Brigham and Women’s Hospital, Boston, MA 02115, USA; 2 Harvard Medical School, Boston, MA 02115, USA; 3 Harvard Humanitarian Initiative, Cambridge, MA 02138, USA; 4 Ministry of Health and Social Assistance, Santo Domingo 10514, Dominican Republic; cecilia.then@ministeriodesalud.gob.do (C.T.P.); ronald.skewes@ministeriodesalud.gob.do (R.S.-R.); 5 Pedro Henríquez Ureña National University, Santo Domingo 10514, Dominican Republic; dukew23@yahoo.com; 6 Department of Infectious Disease Epidemiology and Dynamics, London School of Hygiene & Tropical Medicine, London WC1E 7HT, UK; adam.kucharski@lshtm.ac.uk; 7 University of Queensland Centre of Clinical Research (UQCCR), The University of Queensland, Brisbane, QLD 4029, Australia; colleen.lau@uq.edu.au

**Keywords:** leptospirosis, microscopic agglutination test (MAT), serogroup, serovar

## Abstract

Leptospirosis is a zoonotic disease caused by *Leptospira* spp., with over 250 serovars classified into 24 serogroups. Control measures depend on understanding serovar-specific epidemiology, yet the microscopic agglutination test (MAT) is only serogroup specific, and classification is complicated by cross-reactivity. While MAT is the reference standard for leptospirosis serodiagnosis and seroepidemiological studies, factors influencing serogroup cross-reactivity remain poorly characterized. We investigated the relationship between age, antibody titer, and serogroup diversity among seropositive individuals in a population-based serosurvey in the Dominican Republic. Between June and October 2021, we conducted a multistage national serosurvey, enrolling 6683 participants across 134 clusters. MAT was performed on sera from 2091 participants in two provinces using a 20-serovar panel. MAT positivity was defined as titers ≥ 1:100. Generalized Additive Models were used to assess associations between age, maximum titer, and serogroup diversity. Of 2091 tested samples, 237 (11.3%) were seropositive. Older individuals and those with higher titers reacted to a greater number of serogroups (*p* = 0.005 and *p* < 0.0001, respectively). However, mean maximum titer decreased with age, suggesting that broader serogroup reactivity in older individuals reflects cumulative exposure rather than higher titers. Maximum titer was the strongest predictor of serogroup breadth, while gender, study region, and urban/rural setting were not significant. Overall, our findings demonstrate that serogroup cross-reactivity in MAT was significantly influenced by antibody titer and prior exposure, with older individuals displaying broader serogroup reactivity despite lower titers. These findings highlight key considerations for interpreting MAT results in seroepidemiological studies and underscore the limitations of MAT in serogroup-level classification.

## 1. Introduction

Leptospirosis is caused by pathogenic spirochete bacteria of the genus *Leptospira*, classified into over 250 serovars and 24 serogroups based on their antigenic properties [1]. Different serovars are associated with varying clinical features and disease severities, and are linked to different mammalian reservoirs, including various wild, domestic, and farm animals [2]. Consequently, while leptospirosis is considered a single disease, its clinical manifestations, epidemiology, and transmission pathways are highly variable depending on the infecting serovar. For public health control, this variability has key implications. For example, control measures for *Leptospira interrogans* serovar Icterohaemorrhagiae, primarily transmitted by rats, focus on rodent control. In contrast, for *Leptospira interrogans* serovar Canicola, for which dogs are the primary reservoir, dog vaccination may be the most effective intervention. Therefore, understanding the serovar or serogroup-specific epidemiology in a particular setting is key to inform targeted public health interventions.

The microscopic agglutination test (MAT) is widely used for diagnosing acute leptospirosis infection and conducting population-based seroepidemiological studies. Although MAT has served as the reference standard leptospirosis immunoassay for nearly a century, interpreting results can be challenging. MAT typically uses a panel of around 20 serovars, with reactivity assessed through a dilutional approach to measure serovar-specific titers. Due to frequent cross-reactivity among serovars, results are generally reported at the serogroup level, although serogroup cross-reactivity also remains a challenge. While the factors driving cross-reactivity are not well characterized, they may hold significant implications for result interpretation. In this study, we conduct a secondary analysis of data from a multistage household serological survey in the Dominican Republic [3,4] to examine factors influencing the breadth of MAT-measured serovar and serogroup reactivity.

## 2. Materials and Methods

### 2.1. Study Design, Participant Selection, and Ethical Approvals

As previously described [3,4], between 30 June and 10 October 2021, we conducted a three-stage cross-sectional national household serological survey and enrolled 6683 participants from 3832 households in 134 clusters across all 32 provinces in the Dominican Republic. Of these, sera from clusters in Espaillat province (northwest, *n* = 10) and San Pedro de Macoris province (southeast, *n* = 13) were tested using MAT to detect *Leptospira* antibodies [5,6]. These provinces were intentionally oversampled during the national survey to allow for more granular provincial analyses, given they were the sites of ongoing enhanced prospective acute febrile infection surveillance [7]. Written consent was obtained for all participants. For children < 18 years old, except emancipated minors, consent was obtained from the legal guardian. Written assent was provided by adolescents 14–17 years old, and verbal assent by children 7–13 years old. The study protocol was approved by the National Council of Bioethics in Health, Santo Domingo (013-2019), the Institutional Review Board of Pedro Henríquez Ureña National University, Santo Domingo, and the Mass General Brigham Human Research Committee, Boston, USA (2019P000094).

### 2.2. Immunoassay Characteristics

MATs were used to detect anti-*Leptospira* antibodies and determine the putative serogroups associated with infections. Serological analyses were performed at the US CDC’s Zoonoses and Select Agent Laboratory, Bacterial Special Pathogens Branch, Atlanta, USA. A panel of 20 pathogenic serovars comprising 17 serogroups were selected for the MAT panel (Supplementary Table S1), and samples were tested at dilutions from 1:100 to endpoint. MAT titers ≥ 1:100 were considered seropositive and indicative of prior infection.

### 2.3. Statistical Analyses

We first assessed the association between maximum MAT titers and the number of reactive serogroups. Maximum titer was defined as the highest titer recorded, irrespective of the associated serogroup. To examine the factors affecting the diversity of serogroups and serovars among seropositive cases, we performed partial effects analyses. We applied a Generalized Additive Model (GAM) to titers log10-transformed for normalization. Analysis included smoothed terms for age and maximum titer to understand non-linear associations with serogroup diversity. Covariates considered in the initial model included occupation, study region (northwest vs. southeast), urban vs. rural setting, residence in an informal settlement, education, and reported rat exposure. Variables that did not improve model fit were excluded from the final model. To understand if our findings were consistent across spatially discrete regions, we repeated the analyses after stratifying by study region (NW vs. SE). Analyses and data visualization were performed using the R statistical programming language (R version 4.2.3, 1 March 2023) [8], the mgcv package (version 1.9.1) for partial effect analysis and ggplot2 (version 3.5.2) for data visualization [9].

## 3. Results

Of 2124 participants enrolled in the two study provinces during the national serosurvey, 2091 samples (98.4%) were tested for antibodies to *Leptospira* using MAT. Of these, 237 individuals (11.3%) registered agglutinating antibodies and comprise the current study population. Characteristics of the study population are provided in Table S2. The distribution of reactive serogroups, serovars, and MAT strain are detailed in Table S1.

**Figure 1:**
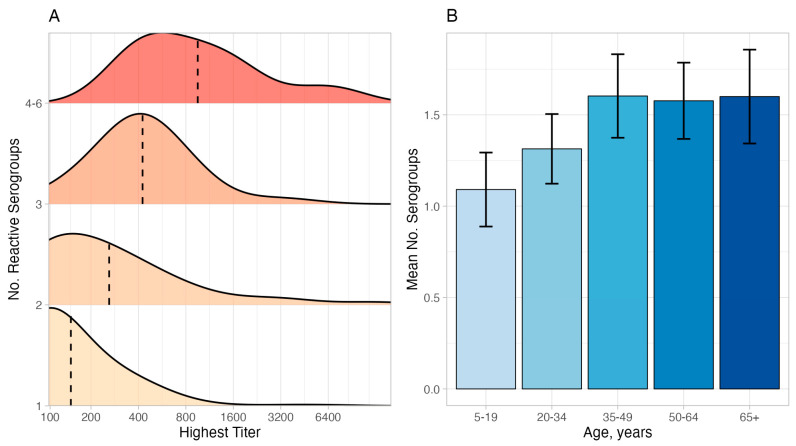
Factors associated with differences in number of reactive *Leptospira* serogroups among seropositive participants, unadjusted, Dominican Republic 2021. (**A**) Ridge plot shows the distribution of the highest titers by the number of reactive serogroups. For example, for sera that reacted to only a single serogroup (lower ridge), the highest titer was 1:100 for most individuals with a smaller number with maximal titers of 1:200, etc. Vertical black lines indicate geometric mean titer. Titers less than 1:100 not measured. N = 237. Titers > 1:12,800 are truncated to improve visualization. (**B**) Mean number of reactive serogroups with 95% confidence intervals stratified by age group. N = 237.

**Figure 2:**
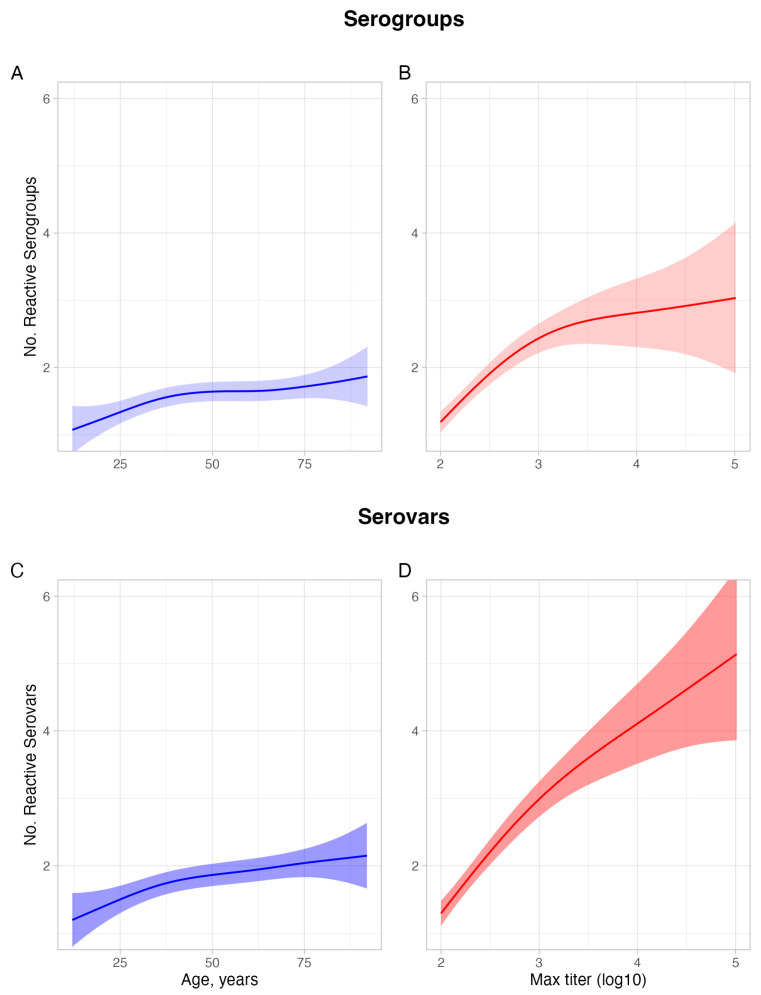
Partial plots of relationship between number of *Leptospira* reactive serogroups (**top row**) and serovars (**bottom row**) by age and maximum titer. Plots (**A**,**B**) show the partial effects of age and log-transformed maximum titer on the number of reactive serogroups in seropositive participants (titers ≥ 1:100), respectively. *p*-value and F-value for the significance of terms were 0.005 and 4.3 for age and <0.0001 and 41.7 for maximum titer, respectively. Plots (**C**,**D**) show the partial effects of age and log-transformed maximum titer on the number of reactive serovars, respectively. *p*-value and F-value for the significance of terms were 0.002 and 5.7 for age and <0.0001 and 61.9 for maximum titer, respectively. Relationships between age and maximum titer were modeled using generalized additive models, with both models accounting for the partial effect of the other explanatory variable. Other potentially relevant variables including gender, study region, occupation, and setting (urban vs. rural) were not associated with number of reacting serogroups or serovars, decreased model fit as assessed by R-squared metric, and therefore are not included in the final models. Colored ribbons represent the 95% CI. N = 237 for all plots.

Sera from older participants and those with higher titers reacted against more serogroups (Figure 1, Table S3). Mean maximum titers, however, trended down among older individuals in both unadjusted and adjusted analyses (Figure S1). Partial effect analyses demonstrated that both age and titer track with the number of reactive serogroups, with independent associations between age and number of reactive serogroups (*p* = 0.005) and maximum titer and number of reactive serogroups (*p* < 0.0001) (Figure 2A,B). Given that we used smoothed predictors in the model, we could not directly compare the relative effect of age versus titer, but given the F-value for maximum titer was ten-fold higher than age (42 vs. 4), we can infer that maximum titer was the most important predictor. Additional variables such as gender, study region, and urban vs. rural settings were not associated with the number of reactive serogroups and reduced model fit when included, so were dropped from the final model. Analysis of serovar rather than serogroup data demonstrated similar trends but, overall, a stronger association between number of reactive serovars and age and titer (Figure 2C,D).

## 4. Discussion

Our findings provide insights into the interpretation of MAT results for leptospirosis, particularly in population-based surveys. We show that higher MAT titers are independently associated with reactivity to a greater number of serogroups, indicating that cross-reactivity increases at high titers but is less pronounced at lower levels. Additionally, while older individuals tend to react to more serogroups, this finding is not driven by higher titers—in fact, titers tend to decline with age. Rather, broader serogroup reactivity in older individuals likely reflects cumulative exposure over time or may involve immunological mechanisms such as memory B cell persistence, affinity maturation, or the accumulation of broadly reactive antibody repertoires [10].

While the challenge of MAT cross-reactivity is well known [11,12], limited investigation has been performed to characterize factors that drive cross-reactivity. Our contribution lies in applying quantitative modeling to a large, population-based serosurvey, enabling us to disentangle the independent and non-linear effects of host age and antibody titer on serogroup breadth. A range of other demographic variables were examined but were not retained in the final models as they did not improve explanatory power or model fit. By using generalized additive models, we were able to move beyond basic correlations and characterize more nuanced associations, including the finding that cross-reactivity increases markedly at high titers but appears to be also influenced by cumulative exposure or potentially immune maturation in older age groups. Our methodological approach provides insights that strengthen the utility of MAT for surveillance and outbreak response, moving beyond the incremental recognition of MAT limitations toward a more robust interpretation of the assay in public health practice. Animal studies have suggested that both the magnitude and breadth of the immune response vary by host species [12], although the role of titer, age, and prior antigen exposure (i.e., immune imprinting) remains unclear. We also found that associations between age or titer and antibody breadth were more pronounced at the serovar level than at the serogroup level. This is not unexpected given serogroups aggregate multiple serovars that—by definition—generate broadly similar immune responses. As such, serovar-level cross-reactivity is more sensitive to differences in age and titer levels, at least in endemic settings.

From a public health perspective, our findings have several implications. First, when designing seroepidemiological surveys, investigators should consider age structure and antibody titers, as cross-reactivity is more likely among these subgroups. Second, in outbreak investigations, the presence of broad serogroup reactivity at high titers should be interpreted cautiously to avoid misattribution of the infecting serogroup. Third, in endemic settings, surveillance programs may benefit from stratifying analyses by age to differentiate recent from cumulative exposures. Lastly, although no widely available human vaccine exists, ongoing efforts to develop region-specific formulations warrant further consideration. Notably, age- and titer-associated patterns of serogroup reactivity may have implications for vaccine development and evaluation. For example, age-specific immune imprinting could influence vaccine responsiveness, underscoring the importance of stratifying future vaccine trials by age to account for age-associated differences in antigenic responses. Taken together, these insights help maximize the utility of MAT data for guiding targeted interventions despite its limitations at the serogroup level.

Beyond human epidemiology, these findings also have One Health implications. MAT is widely used in veterinary and wildlife studies to identify potential reservoirs, yet age- and titer-associated cross-reactivity may equally complicate interpretation in non-human hosts. By clarifying these dynamics in humans, our study underscores the need for integrated approaches that combine human, animal, and environmental data to improve attribution of infection sources. For example, aligning seroepidemiological surveys across species with molecular and ecological investigations could provide more reliable insights into transmission pathways and reservoir contributions.

Our study is limited by the absence of clinical and molecular data, which would have allowed us to directly link serogroup reactivity with infecting strains and disease outcomes—and allow us to assess if increased serogroup reactivity among high titer individuals is primarily driven by recent infection. Future studies would benefit from integrating MAT with molecular methods such as quantitative PCR, multilocus sequence typing (MLST), or whole-genome sequencing (WGS). Such approaches provide higher resolution for distinguishing infecting serovars and linking serological profiles with transmission pathways. Importantly, incorporating intermediate Leptospira species into diagnostic panels may provide additional insights, as these are increasingly recognized as causes of human disease and may contribute to antibody cross-reactivity in MAT assays. Together, these approaches will complement and extend our findings, ensuring that serological insights are more effectively translated into actionable epidemiological and public health knowledge. By using maximum titer as a putative measure of infecting serogroup, this study does not account for serogroup-specific differences in reactivity or differences in host immune imprinting and response. However, although we do report putative infecting serogroup, for additional contextual information (Supplemental Materials), the serogroup specific data does not impact our findings which aimed to assess factors driving cross-reactivity.

## 5. Conclusions

While there are well-recognized limitations to using MAT for serogroup-specific diagnosis, the assay is generally more affordable and sensitive than molecular or culture-based methods and is widely used in both endemic and outbreak settings. As such, studies like ours which help clarify how host factors such as age and antibody titer influence MAT reactivity improve the interpretation of MAT results and maximize its epidemiological utility for public health surveillance and control efforts. By delineating how host age and antibody titers shape MAT cross-reactivity, our study provides practical guidance for improving the design and interpretation of serosurveys, outbreak responses, and surveillance strategies in leptospirosis-endemic regions.

## Data Availability

The original data presented in the study are openly available at https://github.com/enilles1/DR-Leptospirosis/tree/main/data (accessed on 18 September 2025).

## Supplementary Materials

The following supporting information can be downloaded at: https://www.mdpi.com/article/10.3390/tropicalmed10100275/s1, Figure S1. Unadjusted and modeled maximum *Leptospira* titers by age, Dominican Republic 2021. Table S1. Leptospirosis serogroups and serovars among 237 seropositive individuals, Espaillat and San Pedro de Macoris Provinces, Dominican Republic, 2021; Table S2: Characteristics of study population, Espaillat and San Pedro de Macoris Provinces, Dominican Republic, 2021; Table S3. Maximum *Leptospira* titers by number of reactive serogroups, Espaillat and San Pedro de Macoris Provinces, Dominican Republic, July–October 2021.

## References

[1] Palaniappan R.U., Ramanujam S., Chang Y.-F. (2007). Leptospirosis: Pathogenesis, immunity, and diagnosis. Curr. Opin. Infect. Dis..

[2] Pedersen K., Anderson T.D., Maison R.M., Wiscomb G.W., Pipas M.J., Sinnett D.R., Baroch J.A., Gidlewski T. (2018). Leptospira antibodies detected in wildlife in the USA and the US Virgin Islands. J. Wildl. Dis..

[3] Nilles E.J., Paulino C.T., de St Aubin M., Restrepo A.C., Mayfield H., Dumas D., Finch E., Garnier S., Etienne M.C., Iselin L. (2022). SARS-CoV-2 seroprevalence, cumulative infections, and immunity to symptomatic infection—A multistage national household survey and modelling study, Dominican Republic, June–October 2021. Lancet Reg. Health—Am..

[4] Nilles E.J., Paulino C.T., Galloway R., Aubin M.d.S., Mayfield H.J., Restrepo A.C., Dumas D., Garnier S., Etienne M.C., Duke W. (2024). Seroepidemiology of human leptospirosis in the Dominican Republic: A multistage cluster survey, 2021. PLoS Negl. Trop. Dis..

[5] Martin B.M., Sartorius B., Mayfield H.J., Restrepo A.M.C., Kiani B., Paulino C.J.T., Etienne M.C., Skewes-Ramm R., Aubin M.d.S., Dumas D. (2025). Quantifying spatial variation in environmental and sociodemographic drivers of leptospirosis in the Dominican Republic using a geographically weighted regression model. Sci. Rep..

[6] Martin B.M., Sartorius B., Mayfield H.J., Restrepo A.M.C., Kiani B., Paulino C.J.T., Etienne M.C., Skewes-Ramm R., Aubin M.d.S., Dumas D. (2025). Geospatial analysis of leptospirosis clusters and risk factors in two provinces of the Dominican Republic. PLoS Negl. Trop. Dis..

[7] Nilles E.J., Aubin M.d.S., Dumas D., Duke W., Etienne M.C., Abdalla G., Jarolim P., Oasan T., Garnier S., Iihoshi N. (2023). Monitoring Temporal Changes in SARS-CoV-2 Spike Antibody Levels and Variant-Specific Risk for Infection, Dominican Republic, March 2021–August 2022. Emerg. Infect. Dis. J..

[8] R Core Team (2021). R: A language and environment for statistical computing. R Foundation for Statistical Computing.

[9] Wickham H. (2016). ggplot2: Elegant Graphics for Data Analysis.

[10] Syeda M.Z., Hong T., Huang C., Huang W., Mu Q. (2024). B cell memory: From generation to reactivation: A multipronged defense wall against pathogens. Cell Death Discov..

[11] Levett P.N. (2003). Usefulness of serologic analysis as a predictor of the infecting serovar in patients with severe leptospirosis. Clin. Infect. Dis..

[12] Mummah R.O., Gomez A.C.R., Guglielmino A.H., Borremans B., Galloway R.L., Prager K.C., Lloyd-Smith J.O., Chang Y.-F. (2024). Navigating cross-reactivity and host species effects in a serological assay: A case study of the microscopic agglutination test for Leptospira serology. PLoS Negl. Trop. Dis..

