# Supplementary material for: Deciphering Microscopic Agglutination Test (MAT) Serogroup Cross-Reactivity in Leptospirosis: The Influence of Age and Antibody Titers
Source: Trop Med Infect Dis. 2025 Sep 24;10(10):275. doi: 10.3390/tropicalmed10100275 (PMC12567653; doi:10.3390/tropicalmed10100275)
Supplement: Supplementary file 1 [file tropicalmed-10-00275-s001.zip › tropicalmed-3689636-supplementary.pdf]

Supplementary materials

Deciphering MAT Serogroup Cross-Reactivity in Leptospirosis: The Influence of Age and Antibody Titers

Eric J Nilles, et al.

Includes Tables S1-3 and Figure S1

Table S1. Characteristics of study population, Espaillat and San Pedro de Macoris Provinces, Dominican Republic, 2021

Population characteristic	N	No. seropositive	Percent positive
**Overall**	2124	237	11.2
**Gender**			
Female	1339	103	7.7
Male	736	131	17.8
Other	16	3	18.8
**Age cat, years**			
5–19	395	11	2.8
20-34	531	51	9.6
35-49	461	63	13.7
50-64	393	52	13.2
65+	311	60	19.3
**Study region**			
Espaillat	811	127	15.7
San Pedro de Macorís	1280	110	8.6
**Setting**			
Rural	911	128	14.1
Urban	1180	109	9.2
**Reside in barrio**			
No	1527	173	11.3
Yes	541	62	11.5
**Work setting**			
Indoor	175	25	14.3
Outdoor	92	20	21.7
Mix	290	50	17.2
**Education**			
No formal	208	43	20.7
Primary	627	104	16.6
Secondary	772	66	8.5
Technical	44	5	11.4
University	204	15	7.4
**Occupation**			
Farmer	74	26	35.1
Housewife/husband	562	54	9.6
Non-professional	185	29	15.7
Professional	73	9	12.3
Retired	64	14	21.9
Student	420	13	3.1
Other	713	92	12.9

Barrio, unofficial settlement. Missing data includes 25 for Reside in barrio. Work environment enumerated for active workers and excluded students, housepersons, retirees and unemployed. No values were missing for other covariates. Seropositive defined as reactive on MAT ≥1:100.

Table S2. Leptospirosis serogroups and serovars among 237 seropositive individuals, Espaillat and San Pedro de Macoris Provinces, Dominican Republic, 2021

Species	Serogroup	Primary reacting serovar	Strain	No. of positive MATs	Proportion of positive MATs, %
NA	NA	Mixed	NA	42	17.6
L. interrogans	Icterohaemorrhagiae	Mankarso	Mankarso	40	16.8
L. interrogans	Australis	Bratislava	Jez-Bratislava	38	16.0
L. interrogans	Icterohaemorrhagiae	Icterohaemorrhagiae	RGA	38	16.0
L. interrogans	Canicola	Canicola	Ruebush	25	10.5
L. interrogans	Djasiman	Djasiman	Djasiman	19	8.0
L. santarosai	Pyrogenes	Alexi	HS 616	8	3.4
L. interrogans	Pomona	Pomona	Pomona	5	2.1
L. borgpetersenii	Ballum	Ballum	Mus 127	4	1.7
L. borgpetersenii	Tarassovi	Tarassovi	Perepelitsin	4	1.7
L. interrogans	Bataviae	Bataviae	Van Tienen	3	1.3
L. santarosai	Mini	Georgia	LT 117	2	0.8
L. interrogans	Pyrogenes	Pyrogenes	Salinem	3	1.3
L. interrogans	Sejroe	Wolffi	3705	3	1.3
L. interrogans	Autumnalis	Autumnalis	Akiyami A	1	0.4
L. weilii	Celledoni	Celledoni	Celledoni	1	0.4
L. kirschneri	Cynopteri	Cynopteri	3522 C	1	0.4
L. interrogans	Australis	Australis	Ballico	0	0.0
L. santarosai	Hebdomadis	Borincana	HS 622	0	0.0
L. kirschneri	Grippotyphosa	Grippotyphosa	Moskva V	0	0.0
L. borgpetersenii	Javanica	Javanica	Veldrat Bataviae 46	0	0.0
				**237**	**100**

Primary reacting serovar is defined as the serovar that registered the highest titer on microscopic agglutination test (MAT) for each seropositive study participant. Individuals that registered the same highest titer to two or more serovars are categorized as ‘mixed.’ Table previously published at https://journals.plos.org/plosntds/article?id=10.1371/journal.pntd.0012463.

Table S3. Maximum *Leptospira* titers by number of reactive serogroups, Espaillat and San Pedro de Macoris Provinces, Dominican Republic, July-Oct 2021

No. reactive serogroups	N	GMT (95% CI)	Median (IQR)
One	158	149 (134-165)	100 (100, 200)
Two	47	260 (191-355)	200 (100, 400)
Three	23	425 (320-563)	400 (400, 600)
Four to six	8	951 (488-1854)	800 (400, 1600)

Titers measured using the microscopic agglutination test. GMT Geometric mean titers. IQR, interquartile range.

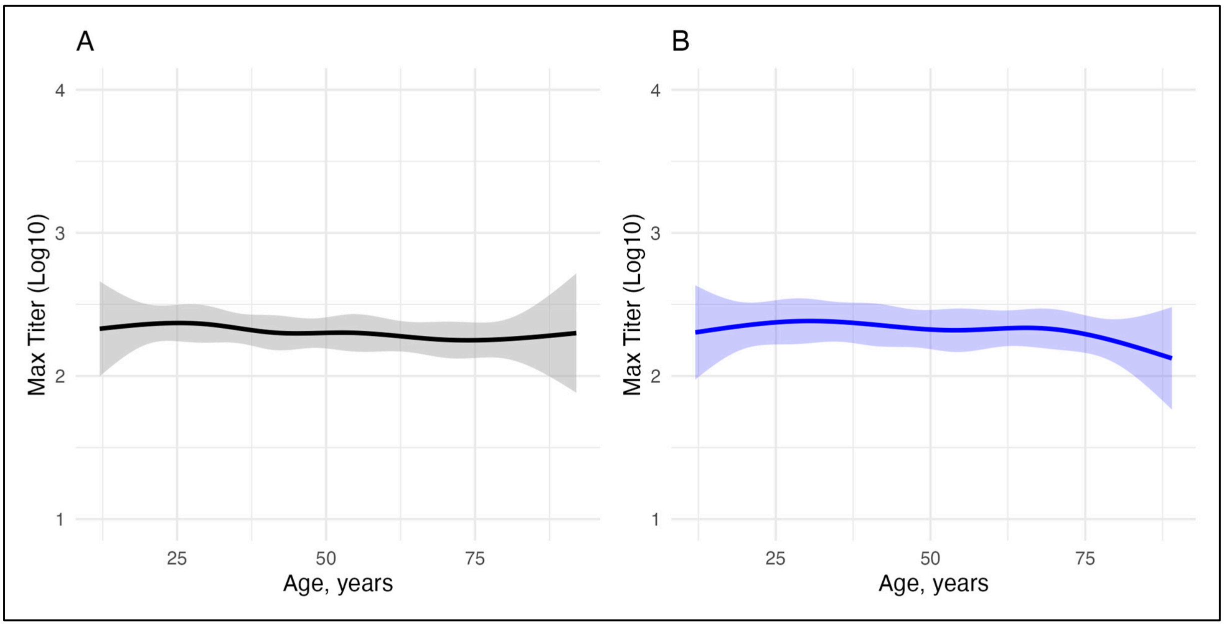

Figure S1. Unadjusted and modeled maximum *Leptospira* titers by age, Dominican Republic 2021. (A) Unadjusted maximum titer by age. **(B)** Multivariable modeled (GAM) maximum titer by age, adjusted for gender, study region (NW vs SE), and setting (rural vs urban). Colored ribbons represent the 95% CI.
